# Effect of physical activity on fasting blood glucose and lipid profile among low income housewives in the MyBFF@home study

**DOI:** 10.1186/s12905-018-0598-9

**Published:** 2018-07-19

**Authors:** Azahadi Omar, Mohd Normazlan Husain, Ahmad Taufik Jamil, Noor Safiza Mohamad Nor, Rashidah Ambak, Mansor Fazliana, Nur Liyana Ahamad Zamri, Tahir Aris

**Affiliations:** 1 Institute for Public Health, National Institutes of Health, Ministry of Health Malaysia, Kuala Lumpur, Malaysia; 2 Department of Community Health, Universiti Kebangsaan Malaysia, Kuala Lumpur, Malaysia; 3 Faculty of Medicine, Universiti Teknologi Mara, UiTM Sg Buloh Campus, Sungai Buloh, Selangor Malaysia; 4 Institute for Medical Research, National Institutes of Health, Ministry of Health Malaysia, Kuala Lumpur, Malaysia

**Keywords:** Physical activity, Blood glucose, Cholesterol, Lipid

## Abstract

**Background:**

Regular physical activity has always been strongly recommended for good cardiovascular health. This study aimed to determine the effect of physical activity on fasting blood glucose and lipid profile among low income housewives in Klang Valley.

**Methods:**

Data of 328 eligible housewives who participated in the MyBFF@Home study was used. Intervention group of 169 subjects were provided with an intervention package which includes physical activity (brisk walking, dumbbell exercise, physical activity diary, group exercise) and 159 subjects in control group received various health seminars. Physical activity level was assessed using short-International Physical Activity Questionnaire. The physical activity level was then re-categorized into 4 categories (active intervention, inactive intervention, active control and inactive control). Physical activity, blood glucose and lipid profile were measured at baseline, 3rd month and 6th month of the study. General Linear Model was used to determine the effect of physical activity on glucose and lipid profile.

**Results:**

At the 6th month, there were 99 subjects in the intervention and 79 control group who had complete data for physical activity. There was no difference on the effect of physical activity on the glucose level and lipid profile except for the Triglycerides level. Both intervention and control groups showed reduction of physical activity level over time.

**Conclusion:**

The effect of physical activity on blood glucose and lipid profile could not be demonstrated possibly due to physical activity in both intervention and control groups showed decreasing trend over time.

## Background

Cardiovascular diseases (CVDs) remain the leading cause of death globally with 17.3 million mortalities per year [1]. Risk factors for the non-communicable diseases (NCDs) are largely preventable such as smoking, poor diet, obesity, physical inactivity and alcohol abuse [2]. Nevertheless, the prevalence of these factors remains on the high side or even increasing in trend in most parts of the world. NCDs are now regarded as the major contributor of preventable disease burden globally, regardless of income [3]. Malaysia is no exception in having to face the same problem of increasing burden of NCDs. National Health and Morbidity Survey (NHMS) 2015 revealed that 17.5% (3.5 million), 30.3% (6.1 million) and 47.7% (9.6 million) of Malaysians age 18 years and above suffer from diabetes mellitus, hypertension and hypercholesterolemia, respectively [4]. A study among urban community found that the NCD problem is more apparent among the poor community [5]. Diabetes mellitus and dyslipidaemia are also important risk factors of developing CVDs. Pre-diabetes status is also associated with increased risk for cardiovascular disease [6]. There is also strong association between dyslipidaemia and CVDs, especially with elevated low-density lipoprotein [7].

Three-quarter of cardiovascular deaths could be prevented by changes in lifestyle including adequate physical activity. There is an inverse dose-response relationship between physical activity and cardiovascular disease and mortality risk. Physical activity can also reduce the impact of cardiovascular diseases, slowing its progress and preventing the recurrence of these diseases [8]. Physical activity and healthy lifestyle have always been strongly recommended for good cardiovascular health [9]. NHMS 2015 also concluded that 66.5% or 14 million of Malaysian adults 18 years or older were physically active, based on the International Physical Activity Questionnaires (IPAQ) definitions [5]. Nevertheless, the figure might have been skewed by the majority involvements of otherwise healthy individuals as has been demonstrated in another study in a different country [10]. Socio-demographic factors have an important influence in determining the participation in physical activity [11]. In a Malaysian study, being a housewife has been shown to be significantly associated with physical inactivity [12]. This inevitably leaves this subgroup of population at higher risk of the consequences of long term physical inactivity including CVDs.

The World Health Organization (WHO) has developed the WHO Global Action Plan for the Prevention and Control of NCDs 2013–2020 to strengthen national efforts in addressing the burden of NCDs [13]. In Malaysia, many activities in the healthy lifestyle programme have been implemented in the past 10 years to prevent obesity and to promote healthy lifestyle among the community. This includes Healthy Eating at Workplace Settings, Healthy Cafeteria, 10,000 steps and healthy weight management by the Ministry of Health, Malaysia. Under a collaborative research framework, My Body is Fit and Fabulous @ Home (MyBFF@home) programme was initiated with multiple objectives including to evaluate the effectiveness of an intervention package in reducing body weight among overweight and obese housewives in Klang Valley and to determine the changes in blood cardio metabolic profiles (glucose, lipid, liver enzymes, obesity related hormones and protein levels) among housewives. The outcome of the study was hoped to present relevant information to support the policymakers in order to enhance the current intervention programme among the female population in Malaysia. This paper focuses on the effect of physical activity intervention on fasting blood glucose and lipid profile among the low income housewives in MyBFF@home study. We hypothesized that the physical activity to be effective in lowering the blood level of fasting blood glucose, total cholesterol, low density lipoprotein level (LDL) and triglycerides whilst increasing the high-density lipoprotein (HDL) level.

## Methods

### Study design and intervention package

This study is part of the ‘My Body is Fit and Fabulous at home (MyBFF@Home) project. MyBFF@Home is a 12-month community-based intervention study. The target population for the project was housewives living in the low-cost flats around Federal Territory of Kuala Lumpur, Klang Valley, Malaysia. Housewives in six flats in the northern area were selected as control groups, while another eight flats in the southern area as intervention groups. The details of the methodology of the study was described elsewhere [14].

The intervention group received specific intervention package for weight reduction including physical activity. The physical activity package includes the following:

i. 15-min brisk walking per day. Those in the intervention group were given OMRON pedometer (Model HJ321) with 7-day memory to monitor their daily steps.
ii. 30-min pillow dumbbell exercise (2 mini dumb bell 300 g) per day. The pillow dumbbells exercise (made from cloth and filled with brown rice with 12 exercise steps) was adapted from Japan [15].
iii. Physical Activity Diary (for self-monitoring tool). The PA diary also contained list of housework chores (moderate to vigorous) and leisure activities based on the Metabolic Equivalence for Compendium List (MET) 2011 [16].

### Blood cardiometabolic profiles

Cardiometabolic profiles assessed in this study were blood glucose and lipid profile (total cholesterol, LDL-cholesterol, HDL-cholesterol and Triglyceride). Following an overnight fast, 12 mililiters of intravenous blood was collected. For glucose measurement, blood samples were collected in fluoride oxalate tubes and centrifuged for 10 min at 3500 rpm for plasma collection. Blood samples for lipid profiles were collected in serum separating tubes, allowed for 30 min to clot at room temperature and centrifuged for 10 min at 3500 rpm for serum collection. Plasma glucose and serum lipid concentration were measured using a biochemistry analyser and assessment were done at baseline, 3rd months and 6th months.

### Physical activity level

The physical activity level assessment was based on the short version of International Physical Activity Questionnaire (IPAQ). The short IPAQ records the activities based on 4 intensity levels (i.e: vigorous, moderate, walking and sitting) with a recall period of ‘last 7-days’ [17]. The analysis of IPAQ was based on the guidelines for data processing and analysis of the IPAQ short version [18]. The respondent was classified as active if they were active at both 3rd month and 6th month of the study. The physical activity level was then re-categorized into 4 categories (active intervention, inactive intervention, active control and inactive control).

### Data management and data analysis

The data analysis plan used various analyses to report the demographic characteristics of housewives and blood profile changes among all 4 physical activity categories. Data analysis was done using Statistical Package for Social Science (SPSS) software version 22. The analyses included descriptive statistics and Analysis of Variance (ANOVA) to compare the baseline difference between groups. General Linear Model (GLM) (i.e: Repeated measures) was used to measure the effect of physical activity on glucose and lipid profile over time. The significance level for this study was set at *p* value of less than 0.05 (*p* < 0.05).

## Results

The study was initiated with 328 participants whom were assigned to the intervention group (*n* = 169) and the control group (*n* = 159). Only 99 participants from the intervention group and 79 participants from the control group had complete data on physical activity for baseline, 3rd months and 6th months of the study. It gave the response rate of 58.6 and 49.7% for both the intervention and the control group. Several reasons for withdrawal of the participants are participants engaged with full time work during the intervention phase, pregnant, and moving out from the area and some personal reasons. Further analysis between respondents and non-respondent showed no significant different between the two groups. The socio-demographic characteristics of the respondents were comparable between these two groups (Table 1).

**Table 1:** Socio-demographic characteristics of respondents after 6 months intervention

Variable	Intervention group *n* (%)	Control group *n* (%)	*P* value
Overall	99	79	
Age group			
18–29	5 (5.1)	6 (7.6)	0.528
30–39	29 (29.3)	16 (20.3)	
40–49	43 (43.4)	39 (49.4)	
50 & above	22 (22.2)	18 (22.8)	
Ethnicity			
Malays	86 (86.9)	74 (93.7)	0.135
Non-Malays	13 (13.1)	5 (6.3)	
Marital status			
Married	87 (87.9)	74 (93.7)	0.191
Unmarried/divorcee	12 (12.1)	5 (6.3)	
Education level			
Primary	16 (16.2)	10 (12.8)	0.533
Secondary/Tertiary	83 (83.8)	68 (87.2)	
Household income			
Less or equal RM 1500	48 (49.5)	27 (35.1)	0.145
RM 1501 – RM 2500	32 (33.0)	29 (37.7)	
RM 2501 – RM 3500	14 (14.4)	14 (18.2)	
More than RM 3500	3 (3.1)	7 (9.1)	
Number of children			
2 or less	21 (21.2)	15 (19.5)	0.841
3–4	47 (47.5)	40 (51.9)	
5 or more	31 (31.3)	22 (28.6)	
BMI (mean,sd)	31.6 (4.35)	31.1 (4.33)	0.364

There were 49 active intervention, 50 inactive intervention, 31 active control and 48 inactive control among the study respondents. Further analysis to compare the socio-demographic characteristic of the 4 groups also showed no different. The baseline fasting blood glucose and lipid profile level is presented in Table 2. The mean baseline fasting blood sugar, total cholesterol, HDL, LDL and triglycerides are also comparable between the two groups.

**Table 2:** Mean blood parameters at baseline

	Intervention Active (Mean, SD)	Intervention Inactive (Mean, SD)	Control Active (Mean, SD)	Control Inactive (Mean, SD)	*P*-value*
FBS, mmol/L	5.3 (0.61)	5.5 (0.97)	5.7 (0.94)	5.8 (1.33)	0.157
TC, mmol/L	5.5 (1.09)	5.7 (0.91)	6.1 (1.08)	5.8 (1.13)	0.154
HDL, mmol/L	1.3 (0.26)	1.3 (0.20)	1.4 (0.22)	1.4 (0.23)	0.117
LDL, mmol/L	4.5 (1.31)	4.4 (0.94)	4.9 (1.24)	4.7 (1.40)	0.441
TG, mmol/L	1.3 (0.63)	1.2 (0.46)	1.6 (0.71)	1.3 (0.64)	0.135

*One-way ANOVA

Table 3 shows the results of physical activity on the blood glucose and lipid profile using General Linear Model analysis. There was no significant effect of physical activity group for blood glucose and lipid profile except for Triglycerides level. Further analysis showed that the significant effect on Triglycerides is among the Intervention Active group and Control Inactive group only (*p* = 0.001). However, the effect size is small (0.08). The MET-minutes per week showed a decreasing trend for all 4 groups.

**Table 3:** Blood glucose and lipid profile by activity levels of intervention and control group

	N	Baseline (Mean, SD)	3 months (Mean, SD)	6 months (Mean, SD)	*P*-value*
Fasting Blood Sugar					
Intervention Active	39	5.4 (0.66)	5.2 (0.53)	5.2 (0.69)	0.596
Intervention Inactive	38	5.5 (1.01)	5.3 (0.73)	5.3 (0.64)	
Control Active	26	5.8 (0.95)	5.5 (0.66)	5.5 (0.89)	
Control Inactive	42	5.8 (1.38)	5.8 (1.60)	5.8 (1.90)	
Total Cholesterol					
Intervention Active	40	5.6 (1.12)	5.5 (0.81)	5.4 (0.96)	0.450
Intervention Inactive	39	5.6 (0.96)	5.3 (0.80)	5.3 (0.98)	
Control Active	28	5.9 (0.99)	5.7 (0.94)	5.8 (1.00)	
Control Inactive	43	5.8 (1.17)	5.4 (1.06)	5.6 (0.98)	
HDL					
Intervention Active	40	1.32 (0.27)	1.34 (0.28)	1.30 (0.29)	0.072
Intervention Inactive	40	1.32 (0.20)	1.31 (0.25)	1.25 (0.25)	
Control Active	28	1.42 (0.20)	1.31 (0.23)	1.32 (0.20)	
Control Inactive	43	1.41 (0.23)	1.32 (0.24)	1.29 (0.24)	
LDL					
Intervention Active	40	4.6 (1.38)	4.4 (0.97)	4.0 (0.98)	0.807
Intervention Inactive	40	4.4 (0.93)	4.2 (0.97)	4.0 (0.96)	
Control Active	28	4.8 (1.19)	4.6 (1.12)	4.2 (0.97)	
Control Inactive	43	4.7 (1.44)	4.4 (1.27)	4.1 (1.04)	
Triglycerides					
Intervention Active	40	1.35 (0.67)	1.54 (0.73)	1.23 (0.50)	0.021
Intervention Inactive	39	1.19 (0.41)	1.40 (0.55)	1.29 (0.45)	
Control Active	28	1.48 (0.63)	1.55 (0.82)	1.38 (0.54)	
Control Inactive	42	1.31 (0.62)	1.39 (0.62)	1.50 (0.66)	
MET-minutes per week					
Intervention Active	49	1598 (2130)	2227 (2072)	1676 (1331)	0.029
Intervention Inactive	49	1722 (2308)	989 (893)	750 (1384)	
Control Active	31	1874 (1883)	1748 (1168)	1455 (1104)	
Control Inactive	46	1694 (2421)	779 (798)	749 (859)	

*GLM (Repeated measures)

## Discussion

At the end of the 6-month period, it was found that the intervention used in this study which includes physical activity did not significantly improve the level of fasting blood glucose or lipid profile of the participants except for triglycerides level only. Few studies had shown the effect of physical activity on triglycerides [19–21]. The finding was inconsistent with other studies which have favourable outcomes in at least one of the components of fasting blood glucose and lipid profile [22–29]. Given the available evidence, we were expecting that the level of fasting blood glucose, total cholesterol or LDL would have some significant decrease in the intervention group, especially among active respondents. Similarly, we were expecting the HDL level to rise significantly in the same group. However, there has been a similar study showing no favourable outcome in fasting blood sugar or lipid profile [30].

This particular outcome could be explained by the total Metabolic Equivalent of Task (MET) minutes per week between the two groups. Interestingly, the total MET minutes for both groups actually decreased between baseline and after 6 months of study. More importantly, there was no difference between the changes of the total MET minutes between the two groups. In another word, during the study period, not only did the intervention group not engage in enough increase in physical activity, they were in fact exercising less. This might be attributed to the fact that the large part of the intervention still required self-initiatives by the individuals in the intervention group. The number of regular formal group physical activities were quite limited during the study period.

The nature or routine of a typical housewife whose primary responsibilities usually resolves around managing daily house chores might hinder them from committing to doing enough exercise [11, 12]. Most of the participants also reported having 3 or more children in the household. This would probably present the housewives with more challenges in committing to physical activity compared their counterparts whom are single or with few or no children.

Even though there were no favourable significant differences in the observed changes of fasting blood glucose and lipid profile between the 2 groups, there were some positives in the outcomes of individual groups. For instance, the total cholesterol level was significantly reduced in both groups, after 6 months. Higher HDL level trend was also seen among those who were active albeit no statistically significant improvement. In another word, the reduction in control group was as good as intervention group to the point where the reduction in intervention group is not superior to the control. The fact that both groups had similar changes at the end of the study might again be explained by the fact that both groups also had similar change of exertion (MET minutes) throughout the study. A similar study with greater emphasize on the amount and compliance of its intervention arm may yield a very different result. The intervention group may need to receive a more regular follow up and more formal sessions to increase the likelihood of adherence to the physical activity regime.

One of the limitations of this present quasi experimental study was the fact that randomisation procedure was not feasible and was not done due to various reasons including logistics issue and limited manpower. Consequently, the subjects recruited in this study might not necessarily reflect the actual population. It is also pertinent to note that the study location means that most of the participants were Malay and thus may not accurately represent all other housewives in Klang Valley. We also had many non-respondents despite various strategies (such as telephone call and home visits) had been employed to increase the response rate. Some of the respondent gave the reason of being busy with the daily work that they couldn’t come during the scheduled follow-ups. Given the nature of the study too, the participants were not overly monitored or controlled as would have been done in a trial. We also didn’t monitor whether the control group gets information from the internet or the clinic that they went. A randomised control trial might have some added value in providing a more definitive conclusion to this intervention study.

## Conclusion

The effect of physical activity intervention in improving the level of fasting blood glucose and lipid profile among the housewives could not be demonstrated as both groups had similar change in the amount of physical activity undertaken as shown by the reduction of MET-minutes per week. This could be due to the programme may also have empowered the knowledge and increased awareness on healthy lifestyle in the control group.

## Abbreviations

ANOVA: Analysis of Varian
CVD: Cardiovascular disease
GLM: General linear model
HDL: High density lipoprotein
IPAQ: International physical activity questionnaire
LDL: Low density lipoprotein
MET: Metabolic equivalent of task
MyBFF@home: My body is fit and fabulous at home
NCD: Non-Communicable disease
NHMS: National health morbidity survey
SPSS: Statistical package for social science
WHO: World Health Organisation
